# Editorial: Women in science - emerging, major & neglected tropical diseases

**DOI:** 10.3389/fitd.2024.1497263

**Published:** 2024-10-17

**Authors:** Yenddy Carrero, Stephanie J. Salyer

**Affiliations:** 1 Universidad Técnica de Ambato, Biociencias Research Group, Ambato, Ecuador; 2 Division of Global Health Protection, Global Health Center, United States Centers for Disease Control and Prevention (US CDC), Atlanta, GA, United States

**Keywords:** women in science, NTDs, neglected tropical diseases, diversity and inclusion, representation

Human-mediated change influencing disease emergence and re-emergence has made this area of research a priority, as these diseases increasingly cause global alarm, impact quality of life, and overwhelm health systems (1). Promoting the involvement of women in science and scientific advancement in the domains of biology, public health, and medicine is crucial today, with a focus on neglected and developing tropical infectious diseases.

Despite the significant contributions women have made to science, societal gender prejudices have prevented women from taking more active roles in the field. Even though this sector has advanced recently and more women are working in it, UNESCO’s Institute for Statistics estimates (only) 33.3% of researchers worldwide are women (2). Several barriers may be limiting the participation and contribution of women in the sciences. While women outnumber male graduates at the university level, men continue to exceed women in most science, technology, engineering, and mathematics (STEM) careers and leadership positions (3, 4). Compared with their male colleagues, women have fewer first-author publications, receive less professional recognition, and are less likely to have research mentorship (5-8). Additionally, female researchers receive fewer grant opportunities and promotions compared with men. Harassment, assault, and marginalization sometimes truncate the careers of promising health scientists, especially those whose race, ethnicity, disability, or sexual orientation make them targets of discrimination (5, 9).

Indeed, many of the researchers involved in efforts to optimize science have recognized a problem centered on gender diversity (10). With the application of gender equality policies, the successful participation of women in research, medicine, and public health has opened a promising avenue for increasing the number of women entering and leading in these fields (11, 12). Changes within the scientific community support more transparent scientific practices and representation, reflecting on historical practices and providing opportunities to address the lack of diversity and non-inclusive culture (13).

Science today benefits from a more collaborative rather than individual process, where team size and participation influence research impact (14). While teams tend to be relatively homogeneous, it is important to proactively address team composition by making them more inclusive and representative of the fields they reflect. Open science, representation, and empowerment are the seeds of a culture of community and sharing and, if cultivated, can continue to foster women’s inclusion and participation.

The important contribution of the first female authors and the growing integration of women in science can be noted in this Emerging and Neglected Tropical Diseases publication series. Highlights of this work include the following studies:

Rodrígues et al. characterized the cellular immune response mediated by Th17 profile cells by *in-situ* determination of the expression of RORγt, IL-17, IL-6, TGF-β, IL-1β, and IL-23 in the clinical–immunopathological spectrum of American cutaneous leishmaniasis (ACL) caused by *Leishmania* (*L.*) *amazonensis* and *Leishmania* (*V.*) *braziliensis*. This study reports that Th17 profile cells could play a significant role in the immunopathogenesis of ACL, through the classical action of Th17 cells and their anti-inflammatory potential. This favors the suppression of the immune response and parasitic persistence in *L.* (*L.*) *amazonensis* with an inflammatory pattern through the “alternative” action of Th17 cells, an exacerbated immune response, parasitic scarcity, and tissue damage in ACL by *L.* (*V.*) *braziliensis*.

Ramkhelawan et al., through a systematic review, found mutations in the β-tubulin gene family of *Ascaris lumbricoides* at codons F200Y (TTC/phenylalanine to TAC/tyrosine), E198A (GAG, GAA/glutamic acid to GCG, and GCA/alanine), and F167Y (TTC, TTT/phenylalanine to TAC, and TAT/tyrosine), which were associated with possible benzimidazole resistance. Knowledge of this resistance further contributes to incorporating approaches for prevention, management, and treatment, thus decreasing the global economic and health burdens of ascariasis due to benzimidazole resistance.

Saboyá-Díaz et al. presented the current neglected infectious diseases situation in the Americas and prospects for their control and elimination as a 2030 goal. They addressed the advances and progress in achieving the objectives of control and elimination of neglected infectious diseases (NTDs) in the Americas and the commitment of the countries to strengthen their capacities and sustain their efforts to achieve the elimination goals.

Chotun et al. addressed the patterns and strategies for neglected tropical disease (NTD) management needed to sustain post-elimination success in African Union member states, as well as the challenges faced, including the potential withdrawal of financial support and the risk of disease re-emergence. This article highlights the importance of continued innovation in surveillance, the critical role of community health workers, the integration of NTD post-elimination strategies into broader health and development frameworks such as universal health coverage, and the need for innovative financing and partnerships to ensure the long-term success of NTD elimination efforts.

The paradigm shift in society regarding the place of women in science has made possible that gender should not be a barrier to research. This, along with the increased demand to address disease resurgence and emergence, makes this a historic moment for women to make a difference. Undoubtedly, the initial steps are the application of better science through more collaborative studies, the promotion of equal opportunities, and the exponential integration of women in research.
